# Evaluating flexure properties, hardness, roughness and microleakage of high-strength injectable dental composite: an in vitro study

**DOI:** 10.1186/s12903-024-04333-3

**Published:** 2024-05-10

**Authors:** Rasha R. Basheer, Fatin A. Hasanain, Dalia A. Abuelenain

**Affiliations:** 1 Restorative Dentistry Department, Operative dentistry division, King Abdulaziz University, Jeddah, Saudi Arabia; 2 Conservative Dentistry Department, Faculty of Dentistry, October University for Modern Sciences and Arts, Giza, Egypt; 3 Restorative Dentistry Department, Biomaterials Division, King Abdulaziz University, Jeddah, Saudi Arabia

**Keywords:** Injectable dental composites, Flexural strength, Hardness, Surface roughness, Microleakage, High strength flowable composites

## Abstract

**Background:**

Recently, a new generation of high-strength flowable dental composites has been introduced by manufacturers. The manufacturers claim that these materials have enhanced mechanical and physical properties and are suitable for use in a wide range of direct anterior and posterior restorations, even in high-stress bearing areas.

**Aim:**

The objective of this study was to assess certain physical and mechanical properties of these recently introduced high-strength flowable composites in comparison to conventional multipurpose dental composites.

**Methods:**

Four types of high-strength flowable composites (Genial Universal FLO, Gaenial Universal Injectable, Beautifil Injectable, and Beautifil Flow Plus) were tested in experimental groups, while a nanohybrid conventional composite (Filtek Z350 XT) was used as the control. For flexure properties, ten rectangular samples (2 × 2 × 25 mm) were prepared from each composite material and subjected to 5000 cycles of thermocycling. Samples were then subjected to flexural strength testing using the universal testing machine. Another twenty disc-shaped specimens of dimensions (5 mm diameter × 2 mm thickness) were fabricated from each composite material for surface roughness (Ra) (*n* = 10) and hardness (VHN) test (*n* = 10). All samples underwent 5000 cycles of thermocycling before testing. Additionally, microleakage testing was conducted on 60 standardized class V cavities prepared on molar teeth and divided randomly into five groups (*n* = 12). Cavities were then filled with composite according to the manufacturer’s instructions and subjected to thermocycling for 1000 cycles before testing using methylene blue solution and a stereomicroscope.

**Results:**

All tested materials were comparable to the control group in terms of flexural strength and surface roughness (*p* > 0.05), with Gaenial Universal FLO exhibiting significantly higher flexural strength compared to the other flowable composite materials tested. However, all tested materials demonstrated significantly lower elastic modulus and surface hardness than the control group (*p* < 0.05). The control group exhibited higher microleakage scores, while the lowest scores were observed in the Gaenial Universal FLO material (*p* < 0.05)

**Conclusion:**

The physical and mechanical behaviors of the different high-strength flowable composites investigated in this study varied. Some of these materials may serve as suitable alternatives to conventional composites in specific applications, emphasizing the importance of dentists being familiar with material properties before making material selections.

## Background

The use of resin-based composite dental materials (RBC) has increased worldwide, because of rising demand for cosmetic, tooth-colored, and mercury-free restorations [1]. Light-cured resin-based dental composite restorative materials are typically used in compound and complex cavities [2, 3]. However, the limited depth of cure of these materials necessitates using multiple incremental layers when placing them in such cavities [4, 5]. Incremental application decreases the shrinkage stress [6] while increasing the time and number of curing cycles required to complete the restoration. As a result, there is a rise in demand from clinicians for RBCs to be provided which use simpler and faster processes.

Some clinicians prefer the use of flowable composite in a large restoration to gain the advantage of using the syringe technique. This method provides easier application in deep or distant areas and better adaptability of flowable composite [7–9]. Flowable composites have been examined in several studies since their introduction to the dental marketplace [8]. Flowable composites are characterized by having lower filler content, which results in lower mechanical properties, higher polymerization shrinkage, lower hardness, lower wear and abrasion resistance [7, 9, 10], and as a result, these materials cannot be used in high stress-bearing areas.

Recently, manufacturers have introduced a new generation of flowable materials with improved mechanical and physical properties which the manufacturers claim can be used in all kinds of direct anterior and posterior restorations, including high-stress bearing areas [11, 12]. However, not enough studies are available to evaluate the suitability of these materials for such applications.

Thus, it is of clinical relevance to evaluate the mechanical and physical properties of different commercially available high-strength flowable dental composites and compare these materials with the more commonly used conventional multipurpose nanohybrid dental composite.

The aim of the present work is to evaluate high-strength flowable composite and compare these materials with the universal multipurpose composite materials regarding flexure strength, elastic modulus, surface hardness, surface roughness, and microleakage.

The first null hypothesis is that there is no significant difference between different types of high-strength flowable composites when compared to conventional nanohybrid composites regarding flexure strength, elastic modulus, surface roughness, and hardness.

The second null hypothesis is that there is no significant difference between different types of high-strength flowable composites when compared to conventional nanohybrid composites regarding microleakage.

## Methods

The present in vitro study was approved by the research ethical committee in King Abdulaziz University Faculty of Dentistry, Jeddah, Saudi Arabia, with ethical number 086–03-23. Materials used in the study are shown in Table 1.

**Table 1:** Details of composite materials and bonding systems used in the study

Group No	Material	Type of Material	Resin component	Filler composition	Filler Weight%; size%	Manufacturer
G1	Filtek Z350 XT	Nanohybrid multipurpose	Bis-GMA, UDMA, TEGDMA, Bis-EMA,	Silica, zirconia Silica/Zirconia nano-clusters	78.5% 5–20 nm	3 M ESPE, St. Paul, MN, USA
G 2	G-aenial universal Flo	Flowable Nano‐hybrid	UDMA, Bis-MEPP, TEGDMA	Silicon Dioxide, Strontium glass	69% 200-nm	GC Corp., Tokyo, Japan
G 3	G-aenial Universal Injectable	Nano-filled High strength Injectable Flowable	UDMA, Bis-EMA, Methacrylate monomers,	Silica, Barium glass	69% 150 nm	GC Corp., Tokyo, Japan
G 4	Beautifil Injectable X	Injectable Bioactive Giomer	Bis-GMA, TEGDMA, Bis-MPEPP,	S-PRG fillers based on aluminofluoroborosilicate glass, Al_2_O_3_	64% 0.8 μm	Shofu Inc., Kyoto, Japan
G 5	Beautifil Flow Plus x F00	Bioactive flowable Giomer	Bis-GMA TEGDMA	S-PRG fillers based on aluminofluoroborosilicate glass, Al_2_O_3_	60% 0.8 μm	Shofu Inc., Kyoto, Japan
	3M Single Bond Universal	Universal Adhesive	MDP Phosphate Monomer, HEMA, Vitrebond Copolymer,	Dimethacrylate resins filler		3 M ESPE, St. Paul, MN, USA
	G-Premio Bond	Universal Adhesive	4-MET, phosphate monomer, Thiophosphate monomer, dimethacrylate,	Fine powdered silica		GC Corp., Tokyo, Japan
	BeautiBond Universal	Self-Etch Adhesive	Phosphonic acid monomer, carboxylic acid monomer, Bis-GMA, TEG-DMA, acetone, water, initiators			Shofu Inc., Kyoto, Japan
	Scotchbond Universal Etchant		32% Phosphoric acid			3 M ESPE, St. Paul, MN, USA

*Bis-GMA* Bisphenolglycidyl methacrylate, *Bis-EMA* Ethoxylated bisphenol-A dimethacrylate, *TEGDM* Triethylene glycol dimethacrylate, *UDMA* Urthane dimethacrylate, *Bis-MEPP* Bisphenol-A ethoxylate dimethacrylate, *Bis-MPEPP* 4-methacryloxy polyethoxyphenyl propane

The sample size calculation was done by considering an alpha value of 0.05 and 80% power to detect a difference of 25%. Considering a common standard deviation of 18% within a single group, the estimated minimum sample size per group should be 10 samples or more [13].

### Sample preparation and testing for flexural strength and elastic modulus

A total of 50 specimens (10 from each group) were constructed from the tested materials in accordance with International Standards Organization (ISO) 4049 specifications for polymer-based restorations [14]. The specimens were all made by the same investigator. A split Teflon mold of dimensions (2 × 2 × 25 mm) was fabricated to prepare the specimens. The mold was placed on a microscope glass slide covered with celluloid strip. The space in the mold was filled with composite resin in one increment. Then, the surface of the restorative materials was covered with another celluloid strip to flatten the surface, then paper clamps were used to secure the glass slabs from both ends. Light curing was done using an LED light curing unit (Bluephase 2: Ivoclar Vivadent, Lichtenstein) with a light intensity of 1200 mW2 for 40 s in three overlapping cycles to cure the 25mm length of the bar from one side only. The light curing unit was tested every 5 specimens with a radiometer (by Demetron, Kerr, USA). Excess material was removed with a sharp scalpel. The dimensions of each sample were measured using a micrometer accurate to 0.01 mm (Mitutoyo digital micrometer, Mitutoyo, Japan). All samples were stored in de-ionized water, in a labeled container for 24 h at 37^0^C. Then samples were subjected to 5000 cycles in a thermocycling machine (1100 SD Mechatronik thermocycler, Westerham, Germany), using distilled water at 5 and 55° C for 30s each. All specimens were subjected to three-point bending using an Instron model 3345 universal testing machine at a span of 20 mm,and a crosshead speed of 0.25 mm/min and 2 KN loading cell. The flexural strength (Mpa), elastic modulus (Gpa), and fracture strain (%) data were calculated and recorded using computer software (BlueHill Universal Instron, England).

### Sample preparation for surface roughness and vickers hardness

For each test, a total of 50 specimens (10 per group) were constructed from the tested materials by the same investigator. A split disc shaped Teflon mold of dimensions (5 mm diameter × 2 mm thickness) was fabricated to prepare the samples. Glass microscope slides, covered with transparent celluloid strips, were positioned at the upper and lower surfaces of the specimen and pressed under hand pressure to extrude excess material. A 500 g weight was placed over the glass slide for one minute to create a flat surface and standardize the force applied to it. Following the removal of the weight, light curing of the specimen was done via the glass slide [15]. LED light curing unit (Bluephase 2: Ivoclar Vivadent, Lichtenstein) with a light intensity of 1200 mW2 for 40 s was used. Excess material was removed with a sharp scalpel. All samples were stored in de-ionized water, in a labeled container for 24 h at 37^0^C.

### Surface roughness testing

Each specimen was fitted to the specimen holder with the surface to be measured and oriented in a horizontal direction. The specimen holder was then moved in a vertical direction up to the specimen surface just touching the measuring tip. Using SJ-210 surface roughness tester (Mitutoyo, Japan). Device calibration was done using the standard calibration specimen before use. The testing parameters were: measuring distance 4 mm, measuring speed 0.5 mm/s, returning 1mm/s, measuring force 0.75 mN, stylus profile: tip radius 2-micron, tip angle 60-degree. The evaluation parameter Ra values were expressed in microns (um).

### Vickers microhardness (VHN) testing

Vickers hardness number (VHN) of each specimen was measured using a microhardness measurement instrument (HMG-G; Shimadzu, Kyoto, Japan). A diamond indenter was used for the microhardness test, and a 100-g load was applied for 10 seconds [16]. Each specimen at the top surface had three indentations made in it, evenly spaced around a circle and at least 1 mm away from the neighboring indentation or specimen boundary. Vickers microhardness number means were determined using the following formula.

$${\text{VHN}}=1.854\mathrm{ P}/{\text{d}}2$$

where 1.854 was a constant value, P was the load applied in (g), d was the diagonal average length in (µm), and HV was the Vickers hardness in (kg/mm2).

### Samples preparation for microleakage test

A total of 60 extracted human molars free of any defects, restorations, or cracks were collected, cleaned, and kept in thymol at 37 °C for not more than a month after. According to the type of material used teeth were randomly divided into 5 groups (*n* = 12). Standardized class V cavities were prepared by the same investigator on buccal surfaces (3 mm width, 3 mm height, and 2 mm depth) above the cementoenamel junction by 1 mm using a high-speed cylindrical 107 μm diamond bur (Komet, Gebr. Brasseler GmbH & Co. KG, Lemgo, Germany). A matrix band with a pre-cut hole 3 × 3 mm was fixed on each tooth with a retainer to aid in the standardization of the cavity outline dimensions. As for the depth of the cavity, it was measured using a premarked periodontal probe. All cavities were prepared with a butt joint by the international guideline, and the margins were not beveled. Prepared cavities in each group were filled with dental composite according to manufacturer instructions using the corresponding adhesive bonding systems as presented in Table 1. LED light curing unit (Bluephase 2: Ivoclar Vivadent, Lichtenstein) with a light intensity of 1200 mW^2^ for 40 s was used. Teeth underwent thermocycling for 1000 cycles in a water bath at 5° and 55°C for 30 s. The entire tooth surface was covered with two layers of nail varnish (essence shine last and go, gel nail polish) within 1 mm of the bonded interface and left undisturbed for one day to allow the varnish to dry. The apices of the roots were sealed using sticky modeling wax (Cavex, Holland). Teeth were immersed in a freshly prepared aqueous methylene blue solution with a concentration of 2 gm/200 cc water for 4 h at room temperature 13. The teeth were vertically sectioned through the center of the restoration, by a cutting machine (IsoMet, 4000 Buehler, Lake Bluff, Illinois, United States) in a buccolingual direction along their long axis to assess the microleakage at the cervical margins. The sections were then separated, and the tooth restoration interface was examined at the cervical margins under a stereomicroscope (Nikon SMZ745T, Tokyo, Japan), at a 40 X magnification interface in which the image of the restoration was captured and transferred to a computer equipped with the image analysis software program (Omnimet, Buehler USA). Four-point scale was used for dye penetration scoring as presented in Table 2 [17].

**Table 2:** Scoring of Microleakage

**Microleakage Score**	**Depth of dye penetration**
0	No dye penetration
1	Dye penetration into half extension of cervical wall
2	Dye penetration into more than half or complete extension of the cervical wall
3	Dye penetration into cervical and axial walls toward the pulp

### Statistical analysis

Data collection and statistical analysis was performed using JMP 17 Statistical Discovery from SAS software (SAS Campus Drive. Cary, NC, USA). The normality of the data was assessed by Shapiro’s test of normality for the five variables (Flexure strength, Elastic modulus, VHN, surface roughness and microleakage scores). Flexure strength and elastic modulus were normally distributed (*p* > 0.05) and three out of the five variables (Hardness, Surface roughness and microleakage scores) produced significant *p*-values for those tests (*p* < 0.05) showing non-normal distribution. Hence, it was decided to use a parametric one way analysis of variance (ANOVA) for flexure strength and elastic modulus and non-parametric tests (Kruskal–Wallis test) for the other three variables for intergroup comparisons.

The results were deemed statistically significant at *p* < 0.05, and Post-hoc Tukey–Kramer tests were used in case of statistically significant difference between groups to delineate areas of significance.

## Results

**Fig. 1:**
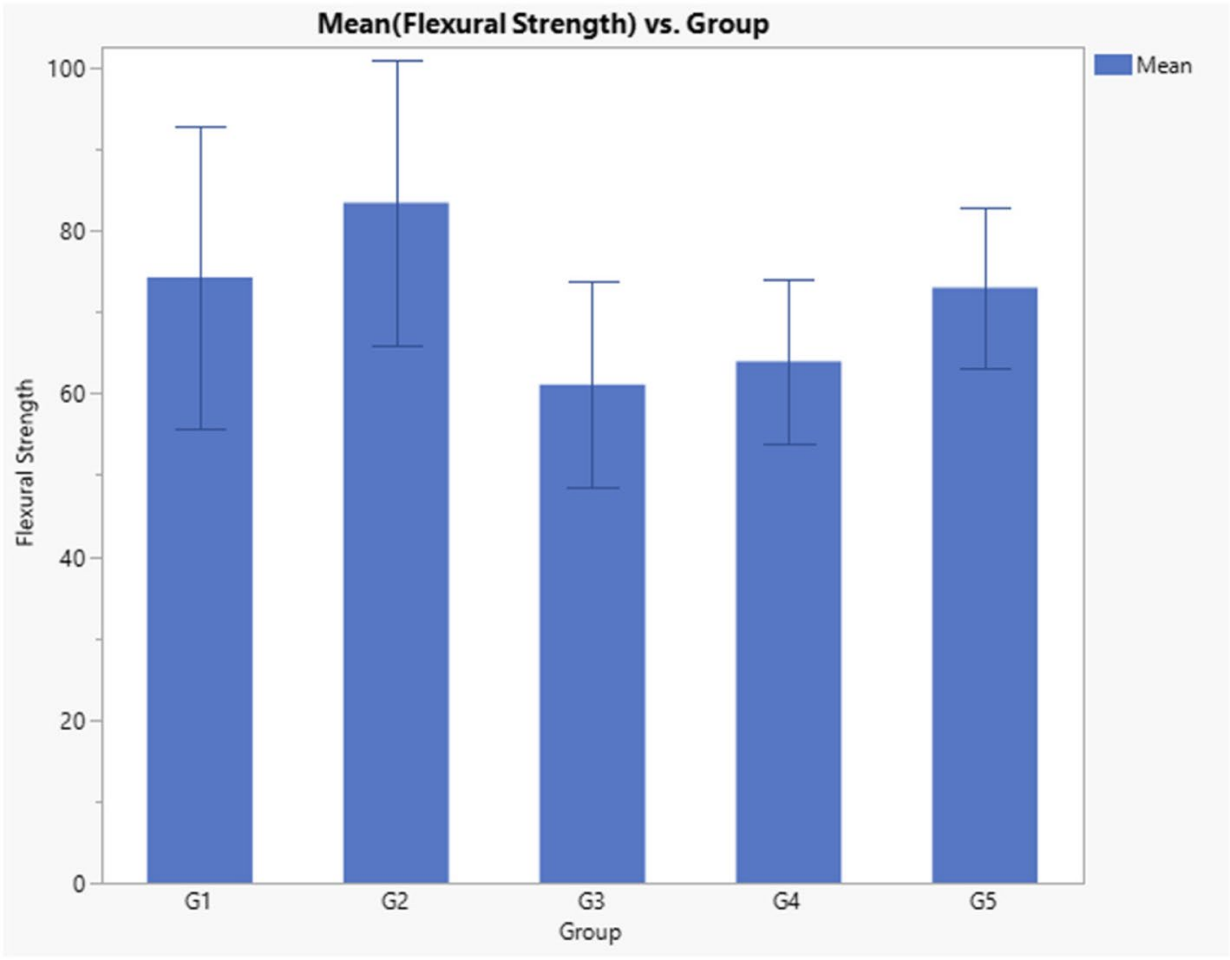
Bar chart comparing the flexure strength (Mpa) of tested composite materials

Results of flexure strength data presented in Table 3 and Fig. 1, indicated that there is a significant difference between groups (*p* = 0.008). Post-hoc test indicated that, when compared to control group (Filtek Z350 XT), there was no statistically significant difference between all tested flowable material compared to the control group (FS 74.25) (*p* > 0.05). However, when comparing the flowable materials, Genial Universal FLO and Beautifil flow plus mean FS values (83.38 and 73, respectively) were significantly higher than Beautifil Injectable and Gaenial universal injectable FS mean values (63.9 and 61.07, respectively) (*p* < 0.05).

**Table 3:** Descriptive statistics and ANOVA *p* Value of Flexure strength data

Group	Number	Mean FS (Mpa)	Std Dev	ANOVA *p* Value
G1(Filtek Z350 XT)	10	74.25^ab^	18.57	0.008*
G2 (Genial Universal FLO)	10	83.38^a^	17.55	
G3 (Gaenial universal injectable)	10	61.07^b^	12.60	
G4 (Beautifil Injectable)	10	63.93^b^	10.11	
G5 (Beautifil flow plus)	10	73.00^ab^	9.87	

Significance level *p*<0.05, * Significant, Groups with different superscript letter are significantly different

**Fig. 2:**
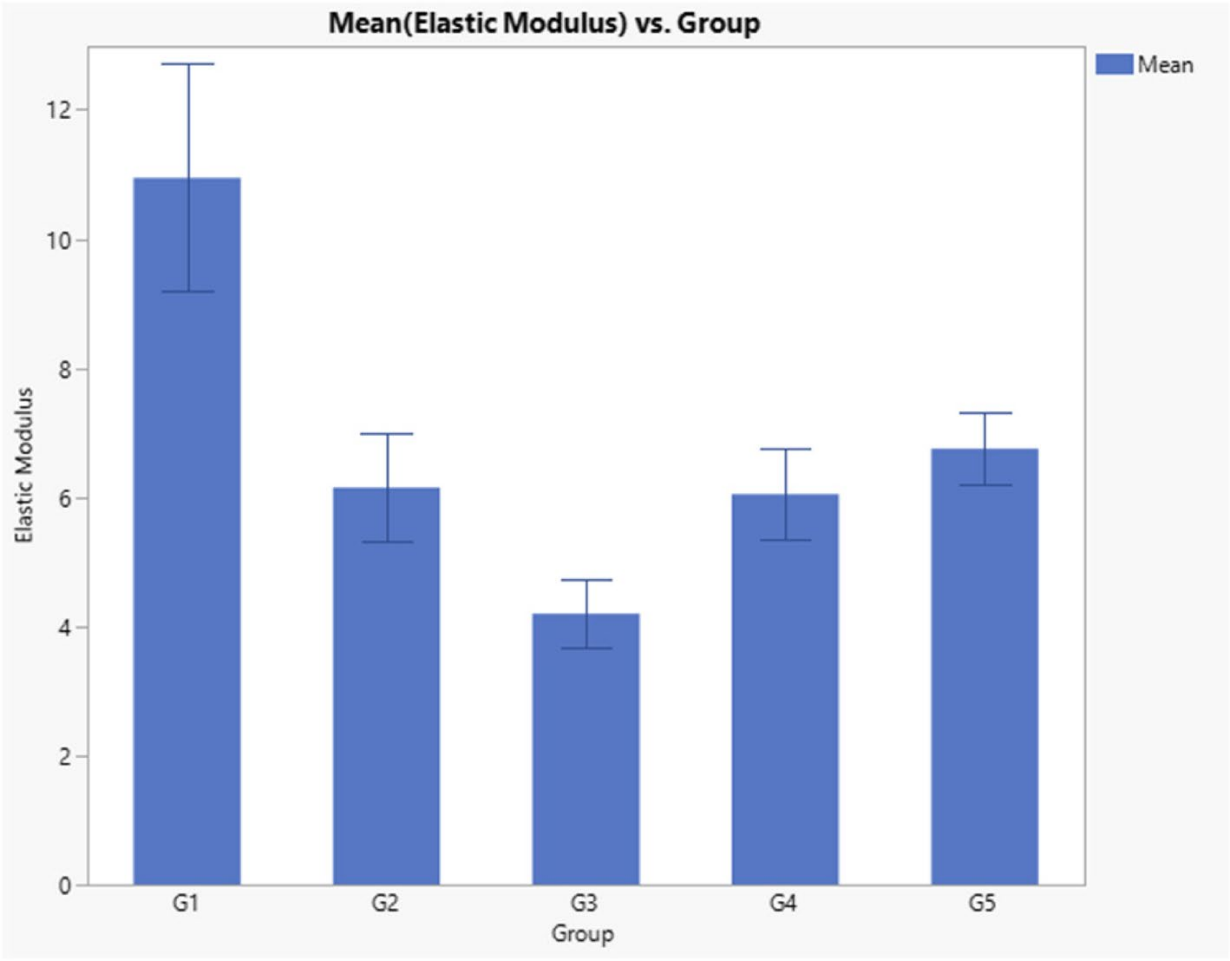
Bar Chart Comparing the Elastic Modulus (Gpa) of tested composite materials

Regarding elastic modulus, results indicated a significant difference between groups (*p* < 0.0001) as presented in Table 4 and Fig. 2. Post-hoc test indicated that Filtek Z350 EM value was significantly higher than other groups (10.94), and Gaenial universal injectable was significantly lower than all other tested groups (4.2). However, there was no significant difference between Beautifil flow plus, Genial Universal FLO and Beautifil Injectable (6.75, 6.14 and 6.05, respectively).

**Table 4:** Descriptive statistics and ANOVA *p* Value of elastic modulus data

Group	Number	Mean Elastic Modulus (Gpa)	Std Dev	ANOVA *p* Value
G1(Filtek Z350 XT)	10	10.94^a^	1.76	< 0.0001*
G2 (Genial Universal FLO)	10	6.14^b^	0.83	
G3 (Gaenial universal injectable)	10	4.2^c^	0.52	
G4 (Beautifil Injectable)	10	6.05^b^	0.71	
G5 (Beautifil flow plus)	10	6.75^b^	0.55	

Significance level *p*<0.05, * Significant, Groups with different superscript letter are significantly different

**Fig. 3:**
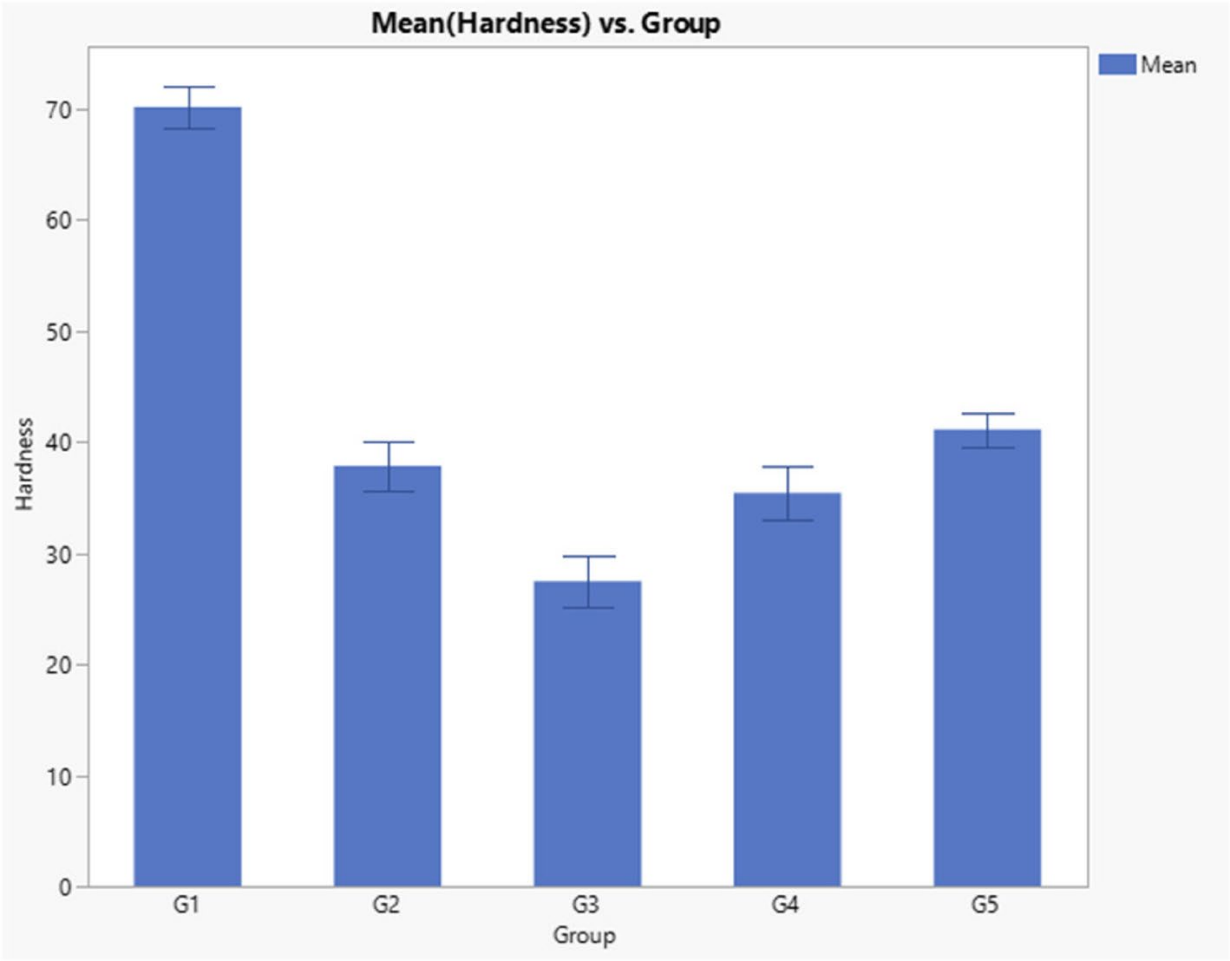
Bar Chart Comparing the hardness (VHN) of tested composite materials

Results of VHN, indicated a significant difference between groups (*p* < 0.0001) as presented in Table 5 and Fig. 3. Post-hoc test indicated that Filtek Z350 XT VHN (70.13) was significantly higher than all other groups and Gaenial universal injectable (27.49) was significantly lower than all other groups, while Beautifil flow plus (41.9) was significantly higher than Genial Universal FLO and Beautifil Injectable ( 37.81 and 35.39, respectively).

**Table 5:** Descriptive statistics and Kruskal–Wallis *p* Value of VHN data

Group	Number	Mean Hardness (VHN)	Std Dev	Kruskal–Wallis *p* Value
G1(Filtek Z350 XT)	10	70.13^a^	1.80	< 0.0001*
G2 (Genial Universal FLO)	10	37.81^c^	2.17	
G3 (Gaenial universal injectable)	10	27.49^d^	2.28	
G4 (Beautifil Injectable)	10	35.39^c^	2.46	
G5 (Beautifil flow plus)	10	41.09^b^	1.57	

Significance level *p*<0.05, * Significant, Groups with different superscript letter are significantly different

**Fig. 4:**
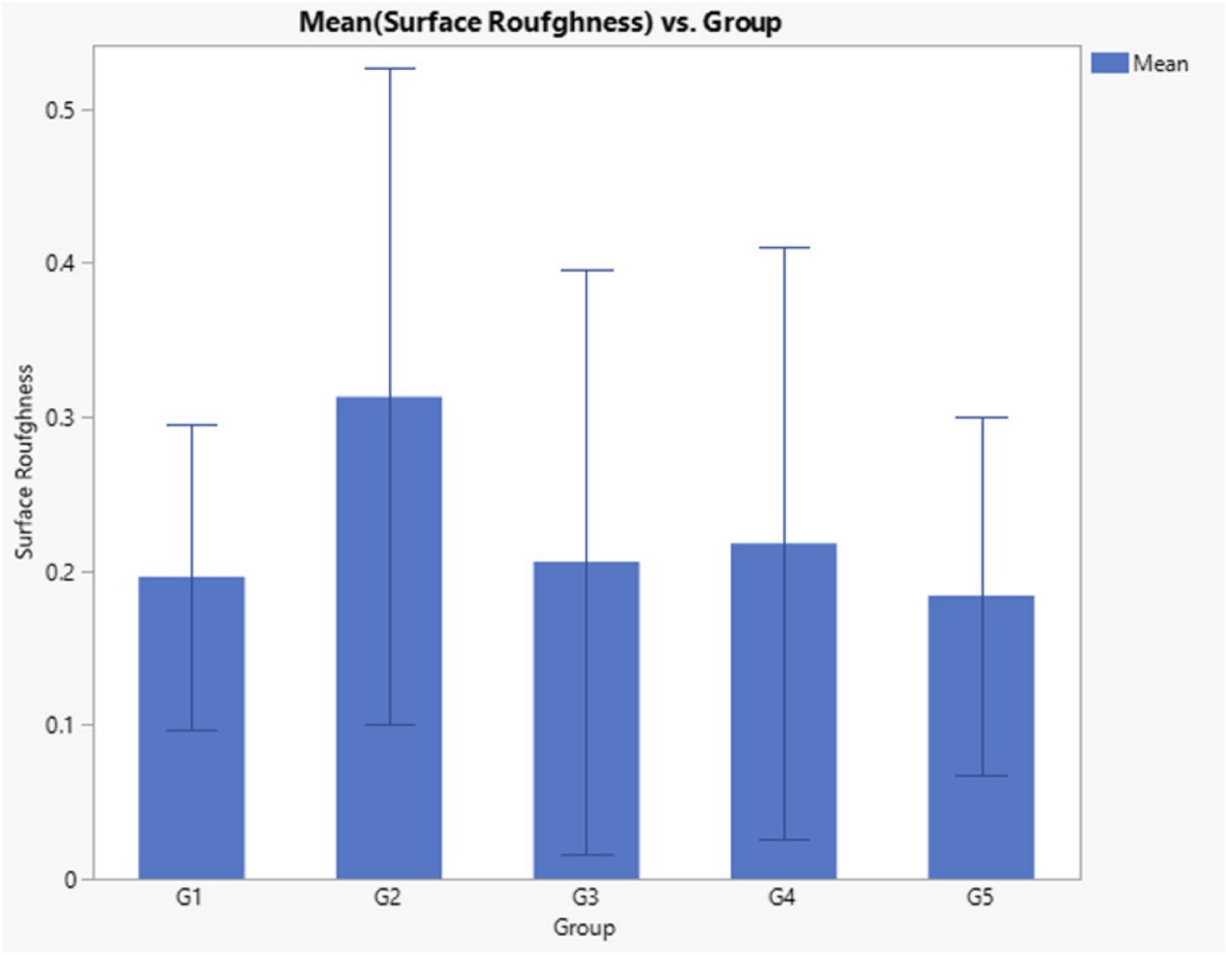
Bar chart comparing the surface roughness (um) of tested composite materials

Regarding surface roughness, results indicated that there was no statistically significant difference observed between test groups (*p* = 0.3) as presented in Table 6 and Fig. 4.

**Table 6:** Descriptive statistics and Kruskal–Wallis *p* Value of surface roughness data

Level	Number	Mean Surface Roughness (um)	Std Dev	Kruskal–Wallis *P* value
G1(Filtek Z350 XT)	10	0.196	0.099	0.3029
G2 (Genial Universal FLO)	10	0.313	0.213	
G3 (Gaenial universal injectable)	10	0.206	0.189	
G4 (Beautifil Injectable)	10	0.218	0.192	
G5 (Beautifil flow plus)	10	0.184	0.116

**Fig. 5:**
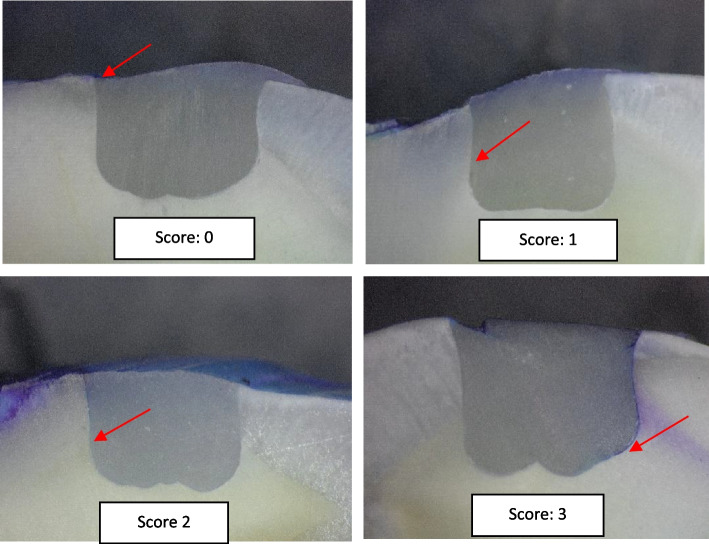
Representative samples showing different scores of microleakage

Results of microleakage scores are presented in Table 7 and, statistical analysis revealed a significant difference between groups (*p* < 0.001) as presented in Table 8. Therefore, the Mann–Whitney test was conducted for pair wise comparison between groups. Compared to the control, there was a significant difference between the control group and groups 2 and 5 (Genial Universal FLO (*p* < 0.001) and Beautifil flow plus (*p* = 0.039)), while the other two group (Gaenial universal injectable (G3) and Beautifil Injectable(G4)) were not significantly different from the control (*p* > 0.05). As presented in Table 7, the control group showed the highest percent of score 3 of microleakage (75%), while all other flowable materials showed lower percent of score 3 with Genial Universal FLO presented the lowest score 3 percent (8%). Representative samples of microleakage are presented in Fig. 5.

**Table 7:** Frequency of microleakage scores (%) in all groups

Group	Score 0	Score 1	Score 2	Score 3	Total
G1(Filtek Z350 XT)	0(0)	0 (0)	3 (25)	9 (75)	12 (100)
G2 (Genial Universal FLO)	6 (50)	4 (33.33)	1 (8.33)	1 (8.33)	12 (100)
G3 (Gaenial universal injectable)	4 (33.33)	1 (8.33)	2 (16.7)	5 (41.7)	12 (100)
G4 (Beautifil Injectable)	0 (0)	1 (8.33)	6 (50)	5 (41.7)	12 (100)
G5 (Beautifil flow plus)	0 (0)	4 (33.33)	4 (33.33)	4 (33.33)	12 (100)

**Table 8:** Descriptive statistics and Kruskal–Wallis *p* Value of Microleakage data

Group	Number	Mean Microleakage Score (%)	Std Dev	Mean Rank	Kruskal–Wallis *P* Value
G1(Filtek Z350 XT)	12	2.75^a^	0.13	43.5	< 0.001*
G2 (Genial Universal FLO)	12	0.75^c^	0.27	14.33	
G3 (Gaenial universal injectable)	12	1.6^abc^	0.39	28.08	
G4 (Beautifil Injectable)	12	2.33^ab^	0.18	35.75	
G5 (Beautifil flow plus)	12	2^b^	0.24	30.83	

Significance level *p*<0.05, * Significant, Groups with different superscript letter are significantly different

## Discussion

The use of resin-based materials is gaining popularity in dental practice due to ease of application, excellent esthetic outcomes, adequate mechanical properties, and adaptability [7, 18]. However, there are still a room for further modification of RBC due to some inherent drawbacks in these materials such as technique sensitivity, polymerization shrinkage which can lead to gap formation and microleakage, time needed for incremental application, low wear and abrasion resistance and marginal deterioration [7, 19].

In the present study, the focus was on evaluating the mechanical and physical properties of four recently introduced high-strength flowable materials. These materials were subjected to thermocycling to simulate aging, and their properties were compared to a widely used conventional nanohybrid posterior composite material, Filtek Z350. Filtek Z350 is routinely employed as a direct restorative material in high strength bearing areas due to its well-documented superior physical and mechanical properties [7].

Thermocycling is a common practice in in vitro studies of dental materials to simulate temperature fluctuations that occur in the oral cavity. This process involves subjecting prepared samples to cycles between 5°C and 55°C, with a dwell time of 30 s [20]. In the present study, a total of 5000 cycles were chosen to represent 500 days of aging in the oral cavity, as supported by existing literature [21].

Flexural strength is a critical parameter used to assess the structural reliability of composite materials and their ability to withstand occlusal loads without fracturing. In addition to that, the flexural test also provides the elastic modulus of the material, which is a measure of the material's rigidity and its ability to withstand occlusal forces without experiencing significant elastic deformation [22], therefore, flexure strength and elastic modulus were conducted in the present study to evaluate the mechanical behavior of the tested materials. Flexure strength and elastic modulus are influenced by several factors in composite materials, including type of resin matrix, degree of crosslinking and polymerization, in addition to type, size and amount of filler loading [12, 23, 24].

The results of the present study demonstrated that all the investigated materials exhibited similar FS when compared to the control group, with some variations observed among the flowable materials. Notably, Genial Universal FLO and Beautifil Flow Plus demonstrated higher FS compared to the other flowable materials. However, when comparing EM, all high-strength flowable materials were significantly lower than the control group (Filtek Z350) and one of these materials (Gaenial universal injectable) was significantly lower than all studied materials.

This disparity can be attributed to the distinct resin combinations and filler technologies employed in these materials. As observed in Table 1, there is a variation in the resin components of all tested materials and variations in the filler loading and fillers type with the two materials provided by Shofu Inc. are considered as giomers because of the presence of aluminofluoroborosilicate and Al_2_O_2_ particles, while the other tested composite materials provided by GC Corp. contains silica, strontium and barium glass particles.

Filtek Z350 XT showed a statistically significant high EM compared to the other tested flowable materials, and this can be attributed to the higher filler loading (78.5% by weight) and the presence of the resin combination of Bis-GMA, UDMA. TEGDMA and Bis-EMA. In which Bis-GMA is a high molecular weight di-methacrylate monomer known to influence the mechanical and physical properties of resin material and provide high EM [25]. Previous studies confirmed that, increased filler content has a positive influence on flexure properties [26] and this goes along with present results. In addition, the result of the current study was following previous work, in which Genial Universal FLO showed significantly high flexure properties when compared to different conventional flowable composite [12].

All flowable tested materials were marketed as a high-strength injectable restorative composite with Genial Universal FLO having a 69% by-weight filler loading of ultra-fine Strontium particles and Gaenial universal injectable as well having a 69% by-weight filler loading of ultra-fine barium particles [27]. Despite the similar filler loading, the two materials are showing different mechanical and physical performances in the current study. On the other hand, Beautifil Injectable was filled with 64% by weight and Beautifil flow plus filled with 60% by weight filler loading, both fillers are based on aluminofluoro-borosilicate glass and Al_2_O_3_. The difference in the filler type and weight percent used in tested materials may explain the difference between the behavior of these materials, as each filler material has different mechanical and physical properties and may influence material behavior differently [27]. This is in addition to the differences observed in the resin matrix of both materials which also contribute to different materials properties.

Surface hardness is a crucial characteristic that indicates a material's resistance to plastic deformation and abrasion [24, 27]. This property is influenced by various factors, including the type of resin matrix and the degree of cross-linking during the polymerization reaction. Furthermore, the surface hardness of composite materials is also affected by the type, size, and volume of filler content [27]. It is worth noting that materials with a higher degree of cross-linking and a greater filler content tend to exhibit enhanced surface hardness [24].

The results of VHN in this study were found to be somewhat similar to the results of the EM test. These findings align with previous studies that suggest materials with high EM values tend to exhibit high surface hardness [24]. In line with this, the present study observed that Filtek Z350, which had significantly higher EM, demonstrated greater surface hardness. On the other hand, Gaenial Universal Injectable, which had significantly lower EM, exhibited lower surface hardness.

Previous studies have established that the surface roughness of dental materials plays a significant role in promoting biofilm formation and plaque accumulation. This, in turn, leads to surface deterioration and degradation of the restoration surface, ultimately facilitating bacterial colonization and the development of recurrent caries over the long term [28]. Therefore, in the present study, surface roughness was assessed to determine the initial surface quality of the materials under investigation and to ascertain whether any of the materials exhibited inherent roughness that could potentially impact biofilm formation and plaque accumulation. Interestingly, the results indicated that there was no significant difference between the examined groups and the control group in terms of surface roughness. This outcome was anticipated since the surface roughness measurements were taken after curing using a celluloid strip and thermocycling, without any additional intervention such as finishing or polishing. The intention was to measure the baseline roughness of the materials. However, it is advisable for future studies to investigate the surface quality after finishing and polishing, as well as after simulating clinical wear and abrasion on the restoration surface.

Based on the previous discussion, the results of the present study reject the first null hypothesis, as there was a significant difference observed between the conventional nanohybrid composite and the studied injectable materials in terms of EM and VHN.

Regarding microleakage, the results obtained indicated that the control group exhibited the highest percentage of score 3 microleakage (50%). In contrast, the Genial Universal FLO composite material demonstrated the lowest percentage of microleakage, with 50% scoring zero and only 8% scoring 3. Possible explanation could be the variation in EM of these materials, Variation in EM values may influence the material behavior, as higher stiffness indicates tough material that may affect the adaptability of the material during polymerization and bonding to tooth structure and may influence the concentration of stresses generated due to polymerization shrinkage. Since materials with high modulus may generate higher stresses during polymerization and bonding to tooth structure and lead to microleakage as observed in the control group and materials with lower modulus may accommodate the generated stresses during bonding due to higher flexibility of the material and less stresses are generated causing better adaptation to walls [29].

This observation aligns with a previous study that demonstrated how composite materials with high flexural strength and elastic modulus tend to exhibit less adaptation to cavity walls compared to materials with lower values of flexural strength and elastic modulus [30].

This outcome could also be attributed to the resin material combinations utilized in Genial Universal FLO. The unique composition of the material, which includes UDMA, Bis-MEPP, and TEGDMA while excluding Bis-GMA, contributes to its flowable nature. This composition may result in lower polymerization shrinkage stresses and higher flowability, allowing for better adaptation to cavity walls and potentially reducing the occurrence of microleakage.

Both Beautifil Injectable materials and Beautifil Flow Plus demonstrated slightly lower microleakage scores compared to the control group, as indicated in Table 7. However, these scores were still significantly higher than those of Genial Universal FLO. It is worth noting that these two materials had a lower filler content, which could potentially lead to higher polymerization shrinkage stresses during the initial stages of polymerization. This, in turn, may result in reduced adaptation of the material to the cavity walls and an increased likelihood of microleakage.

Additionally, the presence of Bis-GMA resin material in these composites, known for its higher polymerization shrinkage stresses, could also contribute to the observed outcomes. These findings align with previous studies that have shown a correlation between higher initial polymerization shrinkage of flowable composites and lower adaptation to cavity walls [30]. Based on the results of microleakage scores, the second null hypothesis was also rejected as explained previously.

The results of this study have important implications for dental practice. While high strength flowable composites have been marketed as suitable for use in all types of direct restorations, including high stress bearing areas, this study shows that not all these materials have equivalent mechanical and physical properties to the conventional nanohybrid posterior composite (Filtek Z350). Dental practitioners should be aware of these differences when selecting materials for direct restorations and the results of the current research highlight the importance of evaluating the mechanical and physical properties of dental materials before using them in clinical practice.

The significantly lower values of flexural strength, elastic modulus, and surface hardness observed in Gaenial Universal Injectable, in comparison to all the materials studied, suggest that this material may not be appropriate for use in high stress bearing areas. Utilizing Gaenial Universal Injectable in such areas could potentially lead to material failure, including restoration fracture or debonding, which would compromise the longevity of the restoration.

On the other hand, Genial Universal FLO demonstrated high flexural strength along with a lower potential for microleakage. This combination of properties indicates that Genial Universal FLO may be well-suited for certain applications that demand high adaptability, strength, and a lower modulus. For instance, it could be considered for use in abfraction lesions or in combination with multipurpose universal composites. The high adaptability of Genial Universal FLO, coupled with its favorable strength characteristics, makes it a potentially suitable choice for these specific clinical scenarios.

## Conclusions

Within the limitations of this study, the usage of high strength flowable composites must be done with full knowledge of the properties of each material prior to its use. This will ensure the success and longevity of the final restoration.

## Data Availability

The datasets used and/or analysed during the current study are available from the corresponding author on reasonable request.
